# Acupuncture points can be identified as cutaneous neurogenic inflammatory spots

**DOI:** 10.1038/s41598-017-14359-z

**Published:** 2017-11-09

**Authors:** Do-Hee Kim, Yeonhee Ryu, Dae Hyun Hahm, Boo Yong Sohn, Insop Shim, O. Sang Kwon, Suchan Chang, Young Seob Gwak, Min Sun Kim, Jae Hyo Kim, Bong Hyo Lee, Eun Young Jang, Rongjie Zhao, Jin Mo Chung, Chae Ha Yang, Hee Young Kim

**Affiliations:** 1 College of Korean Medicine, Daegu Haany University, Daegu, 42158 Korea; 2 Acupuncture, Moxibustion & Meridian Research Center, Division of Standard Research, Korea Institute of Oriental Medicine, Daejeon, 34054 Korea; 3 Department of Physiology, School of Medicine, Kyung Hee Universirty, Seoul, 02447 Korea; 4 Department of Physiology, School of Medicine, Wonkwang University, Iksan, 54538 Korea; 5 Department of Meridian & Acupoint, College of Korean Medicine, Wonkwang University, Iksan, 54538 Korea; 6 School of Mental Health, Qiqihar Medical University, Qiqihar, 161006 China; 7 Department of Neuroscience and Cell Biology, University of Texas Medical Branch, Galveston, TX 77555 USA

## Abstract

Acupuncture, a traditional medical procedure practised for over 2000 years in Asia, stimulates specific but poorly defined sites called acupoints. To date, no unique anatomical acupoint structures have been found. However, noxious sensory signals from visceral organs produce hypersensitive spots on the skin (neurogenic spots), caused by cutaneous neurogenic inflammation, in the dermatome that overlaps with visceral afferent innervations. Here, we show that an acupoint is one form of neurogenic inflammation on the skin. Various studies have demonstrated that acupoints show mechanical hypersensitivity and have high electrical conductance. Stimulation of acupoints produces needling sensations caused by the activation of small diameter afferent nerve fibres and therapeutic effects on the associated visceral organs, which is likely due to the release of endogenous opioids. The present study provides experimental evidence that neurogenic spots exhibit all the characteristics of the acupoints listed above. In addition, the stimulation of neurogenic spots by electrical, mechanical, or chemical means alleviated pathological conditions in rat colitis and hypertension models via the endogenous opioid system. Our results suggest that acupoints associated with internal organs may be identical to neurogenic inflammatory spots on the skin, which are produced by activation of somatic afferents in abnormal conditions of visceral organs.

## Introduction

Over the last 2000 years, acupuncture has been practised in East Asian countries to relieve a variety of illnesses and is now widely used and accepted all over the world. A key of acupuncture treatment is to stimulate specific but poorly defined sites on or under the skin that called acupuncture points or acupoints^1^. Traditional Chinese medicine (TCM) describes how each acupoint communicates with a specific visceral organ; an acupoint reflects the status of a visceral organ, and visceral disorders can be treated by manipulating acupoints^1,2^. Although considerable effort has been devoted towards the identification of acupoints, the anatomical structures of acupoints are largely unknown. A 1977 study by Melzack *et al*. reported that classical acupoints resemble the properties of trigger points – their distribution within the areas of referred pain and the pain relief produced by stimulation of them, although this issue is currently controversial^3,4^. Based on circumstantial evidence, it is generally accepted that acupoints become hypersensitive under certain pathological conditions of visceral organs^5–7^ and have higher electrical conductance than the surrounding tissue^8,9^. Manual or electrical stimulation of acupoints can relieve the symptoms of the associated visceral organs^1,2^, possibly via endogenous opioid mechanisms^10–12^. Needling of an acupoint generates small diameter nerve fibres-mediated sensations (*Deqi*), crucial for producing the therapeutic effects of acupuncture^13,14^.

Visceral disorders frequently produce a referred pain at topographically distinct body surfaces^15^ due to the convergence of visceral and somatic afferents on the same neuron in the sensory pathway^16^. In multiple sites of skin overlying the referred pain, local tissue responses, known as neurogenic inflammation (neurogenic spots), are observed, which range from 0.5–2 mm in diameter in rats^17^ and can be visualized experimentally in the skin by systemic injection of Evans blue dye^17^. The features of neurogenic spots include plasma extravasation and vasodilation in the postcapillary venules of the skin and wheal-and-flare reaction arising from the release of calcitonin gene-related peptide (CGRP) and substance P (SP) from activated small diameter sensory afferents^18^. Neurogenic spots may show hypersensitivity, high electrical conductance, and small diameter nerve fibres-mediated sensations, similar to the physiological features of acupoints. The present study attempted to show that acupoints have similarities with the neurogenic spots induced by neurogenic inflammation in the dermatome associated with visceral disorders.

## Results

### Comparisons of the anatomic location between neurogenic spots and acupoints

**Figure 1:**
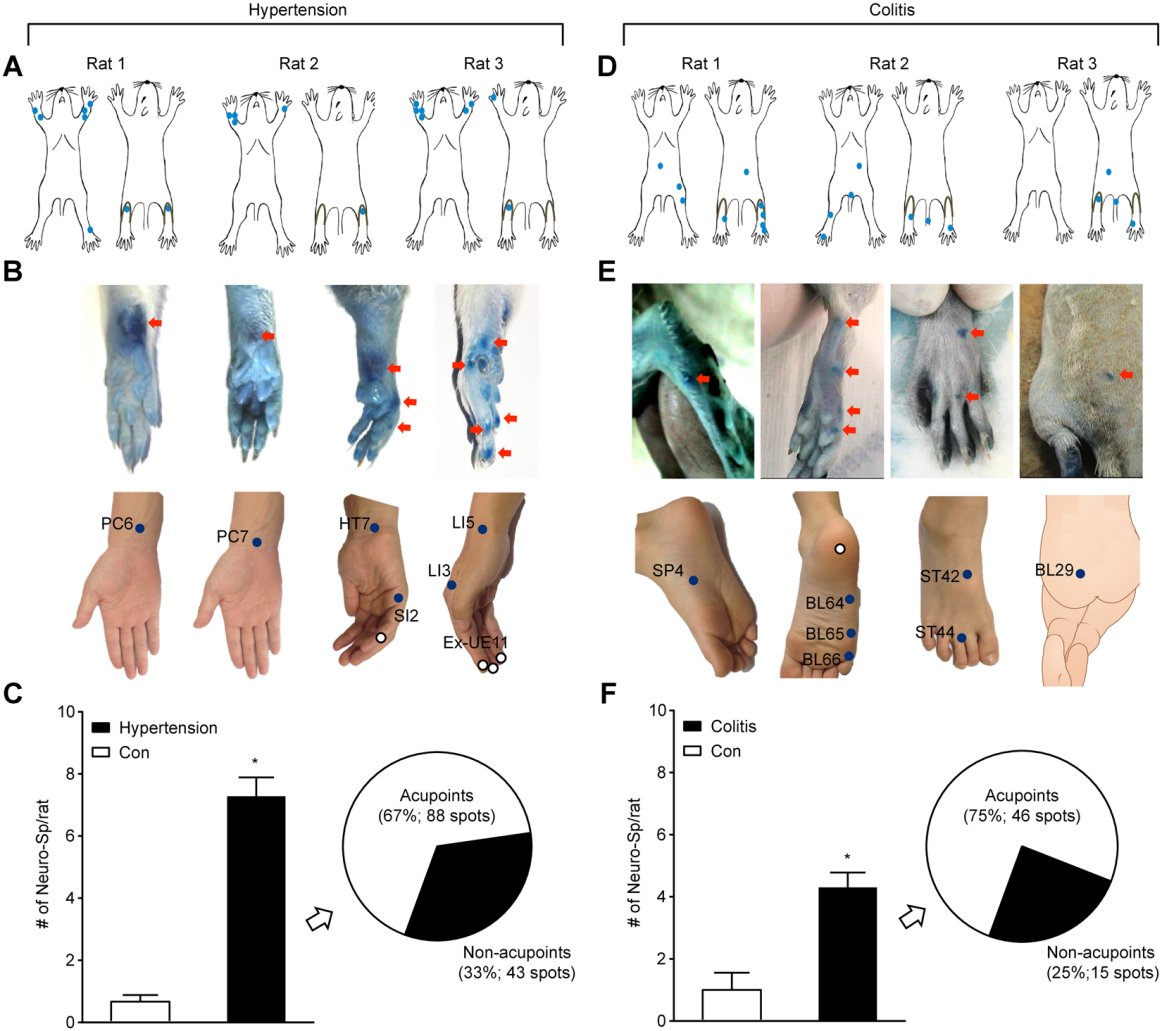
Neurogenic inflammatory areas in the skin (neurogenic spots) anatomically correspond to traditional acupuncture points. (**A**,**D**) Skin distribution of neurogenic spots identified by Evans blue dye in hypertensive (**A**) or colitis rats (**D**). (**B**,**E**) Anatomical correspondence of neurogenic spots and traditional acupoints in hypertensive (**B**) or colitis rats (**E**). Filled-in blue circles and open circles on human skin represent traditional acupoint and non-traditional acupoint, respectively. (**C**,**F**) Numbers of neurogenic spots per animal (bar graphs) and correlation of anatomic location between neurogenic spots and traditional acupoints in hypertensive (n = 18; (**C**) or colitic rats (n = 13; (**F**) (pie graphs). P < 0.001 vs. Con.

As the first step of the investigation, we asked if neurogenic spots were found in the anatomical location of traditional acupoints. Cutaneous neurogenic inflammatory sites (neurogenic spots; Neuro-Sp)^17^ were detected by exploring the leakage of intravenously injected Evans blue dye to the skin in rat models of hypertension or colitis. Then, the neurogenic spots were mapped and compared with the corresponding human anatomical acupoints (Fig. 1A,B,D and E), based on the transpositional method^19^. The blue spots, ranging in diameter from 0.5 mm to 3 mm, started to appear approximately 5–10 min after intravenous injection of Evans blue dye (50 mg/kg) in the rats. The spots were maintained during a 2-hour immobilization in the hypertension model. The spots in the colitis model gradually faded over the next several days. Approximately 7 and 4 spots per animal were observed in the hypertension (Fig. 1C) and colitis (Fig. 1F) models, respectively, whereas very few spots were observed in the control rats. In the hypertensive rats (n = 18), the majority of the neurogenic spots appeared bilaterally or unilaterally on the forelimb (90 of 131 spots), and 67% of those spots matched with acupoints, such as PC6 (28 spots), PC7 (24 spots), and HT7 (22 spots) (Fig. 1B,C and Supplementary Table S1). In the colitis rats (n = 13), 75% of the neurogenic spots (46 of 61 spots) corresponded to acupoints on the hind limb, such as SP4 (12 spots), ST44 (7 spots), and BL66 (4 spots) (Fig. 1E,F and Supplementary Table S1). These results indicate that the majority of the neurogenic spots (70%; 134 of 192 spots) coincided with the location of acupoints.

### Effects of neurogenic spot stimulation on hypertension or colitis

**Figure 2:**
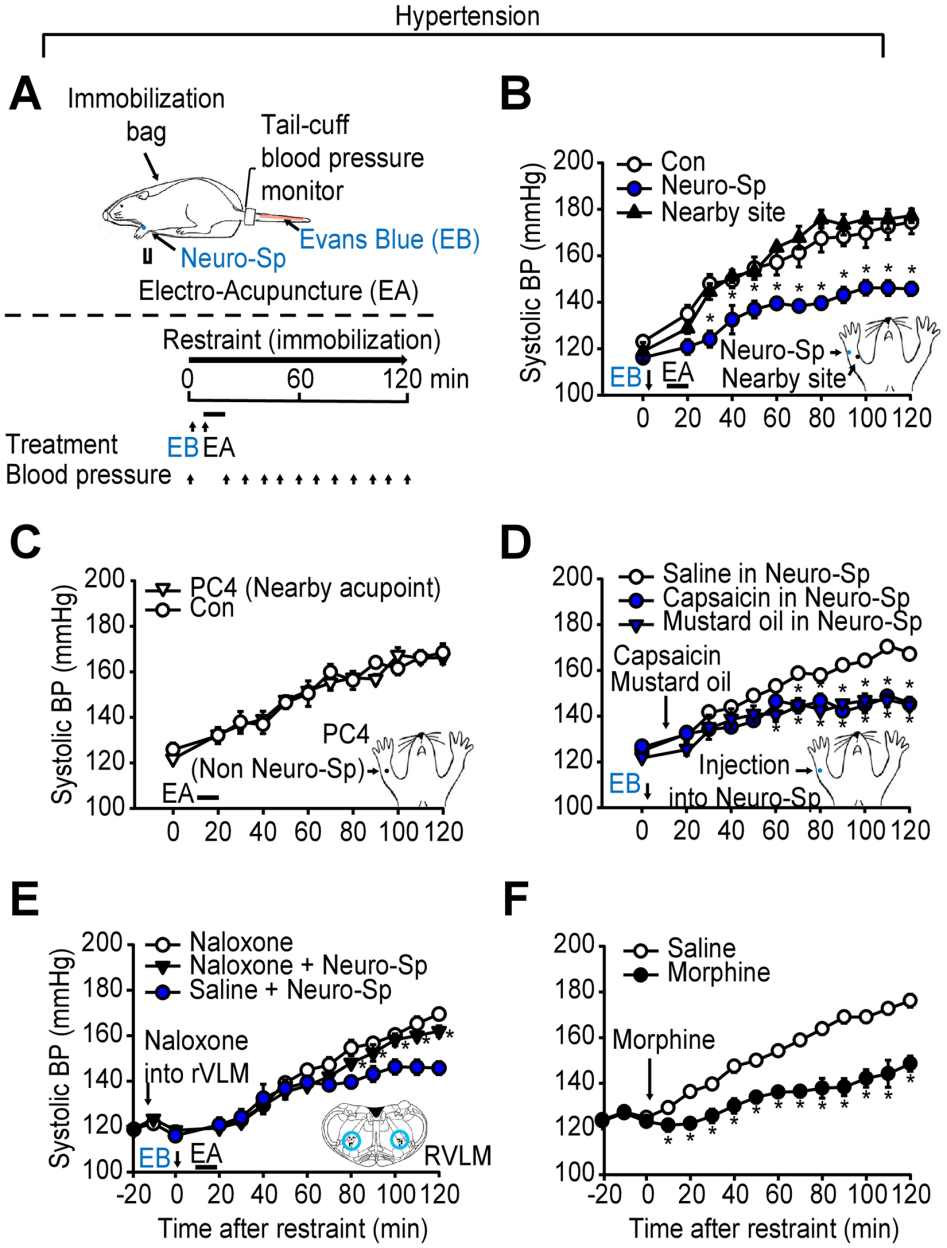
Stimulation of a neurogenic spot prevents the development of hypertension. (**A**) Schematic of the experimental procedure in the hypertension model. Evans blue dye (EB) was injected via the tail vein after the initiation of restraint. Approximately 10 min after EB injection, electroacupuncture (EA) at neurogenic spots (Evans blue dots) on the forelimb was applied for 10 min, and blood pressure was measured every 10 min. (**B**) EA at neurogenic spots prevents the development of hypertension (n = 5–8). *P < 0.05 vs. Con or Nearby site. (**C**) EA at a non-neurogenic acupoint PC4 fails to suppress hypertension (n = 5). (**D**) Injection of capsaicin or mustard oil into a neurogenic spot suppresses the development of hypertension (n = 6), while injection of saline in a neurogenic spot does not affect the development of hypertension (Saline in Neuro-Sp). *P < 0.05 vs. Saline in Neuro-Sp. (**E**) An RVLM injection of naloxone interferes anti-hypertension effects by EA stimulation of neurogenic spot (n = 5), while saline injection into RVLM prior to EA does not affect anti-hypertensive effects of EA (Saline + Neuro-Sp). *P < 0.05 vs. Saline + Neuro-Sp. (**F)** Intraperitoneal injection of morphine (10 mg/kg) prevents the development of hypertension (n = 7). *P < 0.05 vs. Saline.

To explore whether stimulation of neurogenic spots can generate acupuncture-like effects, neurogenic spots were stimulated to determine if they could alleviate the development of hypertension or colitis in rats. When a rat was placed in an immobilization bag, the systolic blood pressure of the rat gradually began to elevate over the next several hours (Fig. 2A,B), as reported previously^20,21^, reaching the level of hypertension (over 150 mmHg) within 1 hour, and lasting until the end of the 2 hr immobilization. The development of hypertension was significantly reduced when electrical acupuncture was applied to neurogenic spots on the palm side of the forelimb (treatment F(2, 8) = 61.573, P < 0.001, Partial Eta-Squared = 0.93; time F(12, 48) = 88.524, P < 0.001; interaction F(24, 96 = 1.680), P = 0.041, Partial Eta-Squared = 0.46; Fig. 2A,B). However, the same stimulation of a nearby site 3–5 mm away from neurogenic spot did not produce the same effect. In addition, stimulation of a non-neurogenic acupoint, PC4, which is used in acupuncture clinics to treat hypertension^1^, did not lower hypertension (treatment, P = 0.73, Partial Eta-Squared = 0.03; Fig. 2C). Furthermore, when capsaicin (a TRPV1 agonist that activates C and Aδ fibres) or mustard oil (a TRPA1 agonist) was injected into the neurogenic spots, the development of hypertension was blocked, which was not observed in the saline-treated group (treatment F(2, 8) = 32.474, P < 0.001, Partial Eta-Squared = 0.890; time F(12, 48) = 38.40, P < 0.001; interaction F(24, 96) = 3.774, P < 0.001, Partial Eta-Squared = 0.485; Fig. 2D). Taken together, these results suggest that the stimulation of neurogenic spots by electrical or chemical means could produce therapeutic effects in the same manner as traditional acupuncture.

Since the role of the endogenous opioid system in the effects of acupoint stimulation has been well recognized^10^, we also tested the possible involvement of the endogenous opioid system in the effects of stimulation of neurogenic spots. Pre-treatment of the RVLM with naloxone (10 nM, 0.1 µl; an opioid antagonist) completely blocked the neurogenic spot stimulation-induced suppression of hypertension (treatment F(2, 8) = 7.559, P = 0.014, Partial Eta-Squared = 0.653; time F(12, 48) = 131.177, P < 0.001; interaction F(24, 96) = 2.212, P = 0.004, Partial Eta-Squared = 0.355; Fig. 2E). The anti-hypertensive effect of neurogenic spot stimulation was mimicked by administration of the opioid agonist morphine (treatment F(1, 6) = 182.240, P < 0.001, Partial Eta-Squared = 0.966; time F(12, 72) = 53.770, P < 0.001; interaction F(12, 61) = 6.684, P < 0.001, Partial Eta-Squared = 0.567; Fig. 2F).

**Figure 3:**
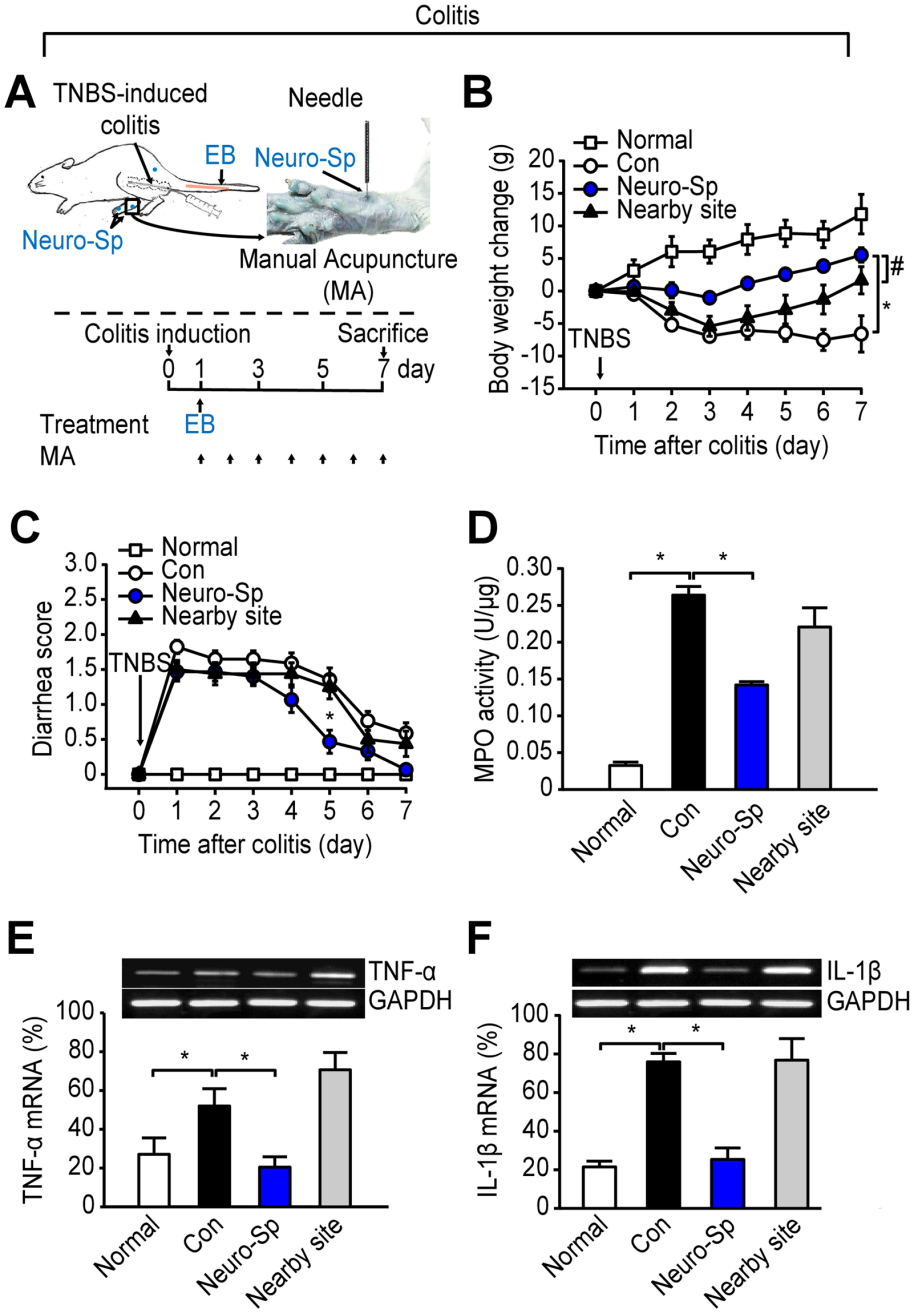
Stimulation of a neurogenic spot attenuates colitis. (**A**) Schematic of the experimental procedure in the colitis model. Approximately 10 min after EB injection, manual acupuncture (MA) at a neurogenic spot on the hind paw was performed once daily for 7 days. MA at neurogenic spots alleviated the body weight changes (**B**) and diarrhea scores (**C**) in the colitis rats and normalized the increased level of MPO activity (**D**) TNF-α (**E**) and IL-1β (**F**) in the colitis rats compared to MA at a nearby site. Normal, normal rats (n = 5); Con, colitis rats (n = 14); Neuro-Sp, acupuncture at neurogenic spot for 7 days (n = 17); Nearby site, acupuncture at nearby site 3–5 mm apart from neurogenic spot (n = 14). *P < 0.05 vs. Con; ^#^P < 0.05 vs. Nearby site.

The acupuncture effects of stimulation of neurogenic spots were further confirmed in the colitis model (Fig. 3A–F). Compared with normal rats (Normal), the intracolonic TNBS-treated rats (Con) showed a slow and sustained decrease in body weight over 7 days and severe bloody diarrhea up to 5 days after the induction of colitis. Rats that received daily manual acupuncture treatment at a neurogenic spot near the base of the 1st metatarsal bone (Neuro-Sp, Fig. 3A) showed partial recovery of the weight loss (treatment F(4, 12) = 5.566, P = 0.013, Partial Eta-Squared = 0.581; time F(7, 28) = 8.390, P = 0.001; interaction F(21, 84) = 1.896, P = 0.021, Partial Eta-Squared = 0.321; Fig. 3B) and diarrhea symptoms (treatment F(3, 27) = 19.859, P < 0.001, Partial Eta-Squared = 0.688; time F(7, 63) = 50.149, P < 0.001; interaction F(21,189) = 10.340, P < 0.001, Partial Eta-Squared = 0.534; Fig. 3C) compared to the control groups. Seven days after colitis induction, the extent of inflammation in the colon was assessed by measuring neutrophil infiltration (MPO activity), proinflammatory cytokine levels (TNF-α and IL-1β), and inflammation parameters. MPO activity (Fig. 3D), the TNF-α level (Fig. 3E), and the IL-1β level (Fig. 3F) in the colitis rats were significantly higher than those in the control rats, and these effects were attenuated by acupuncture treatments at a neurogenic spot (Neuro-Sp; Fig. 3), but not by stimulation of a nearby non-neurogenic site (Nearby site; Fig. 3). In terms of the inflammatory parameters, the adhesion and macroscopic damage scores were lower in the group receiving acupuncture at the neurogenic spots than in the control groups (Table 1). Taken together, the data suggest that acupuncture stimulation at neurogenic spots produces therapeutic effects on the associated visceral disorders.

**Table 1:** Colonic inflammation.

Group	Colon weight/length (mg/cm)	Adhesion (score 0–2)	Diarrhea (score 0–1)	Macroscopic damage (score 0–10)
Normal	111 ± 30	0	0	0
Con	236 ± 36	3.38 ± 0.55*	0.66 ± 0.11*	3.38 ± 0.51*
Neuro-Sp	162 ± 37	0.84 ± 0.27^#^	0.23 ± 0.12	0.84 ± 0.27^#^
Nearby site	182 ± 20	2.5 ± 0.46	0.43 ± 0.12	2.5 ± 0.46

Normal, normal rats (n = 15); Con, colitis rats (n = 20); Neuro-Sp, acupuncture at neurogenic spot for 7 days (n = 20); Nearby site, acupuncture at nearby site 3–5 mm apart from neurogenic spot (n = 20). The values are presented as the means ± S.E.M. One-way ANOVA with post hoc Tukey test. *P < 0.05, Normal vs. Con; ^**#**^P < 0.05, Con vs. Neuro-Sp.

### Measurement of electrical conductance, mechanical sensitivity and CGRP expression on neurogenic spots and a retrograde labelling study

Next, we investigated whether the neurogenic spots showed high electrical conductance, hypersensitivity, small fibre (C & Aδ) mediation in producing the therapeutic effects and a connection with the internal organ, similar to the physiological features of acupoints^7–9,13^.

**Figure 4:**
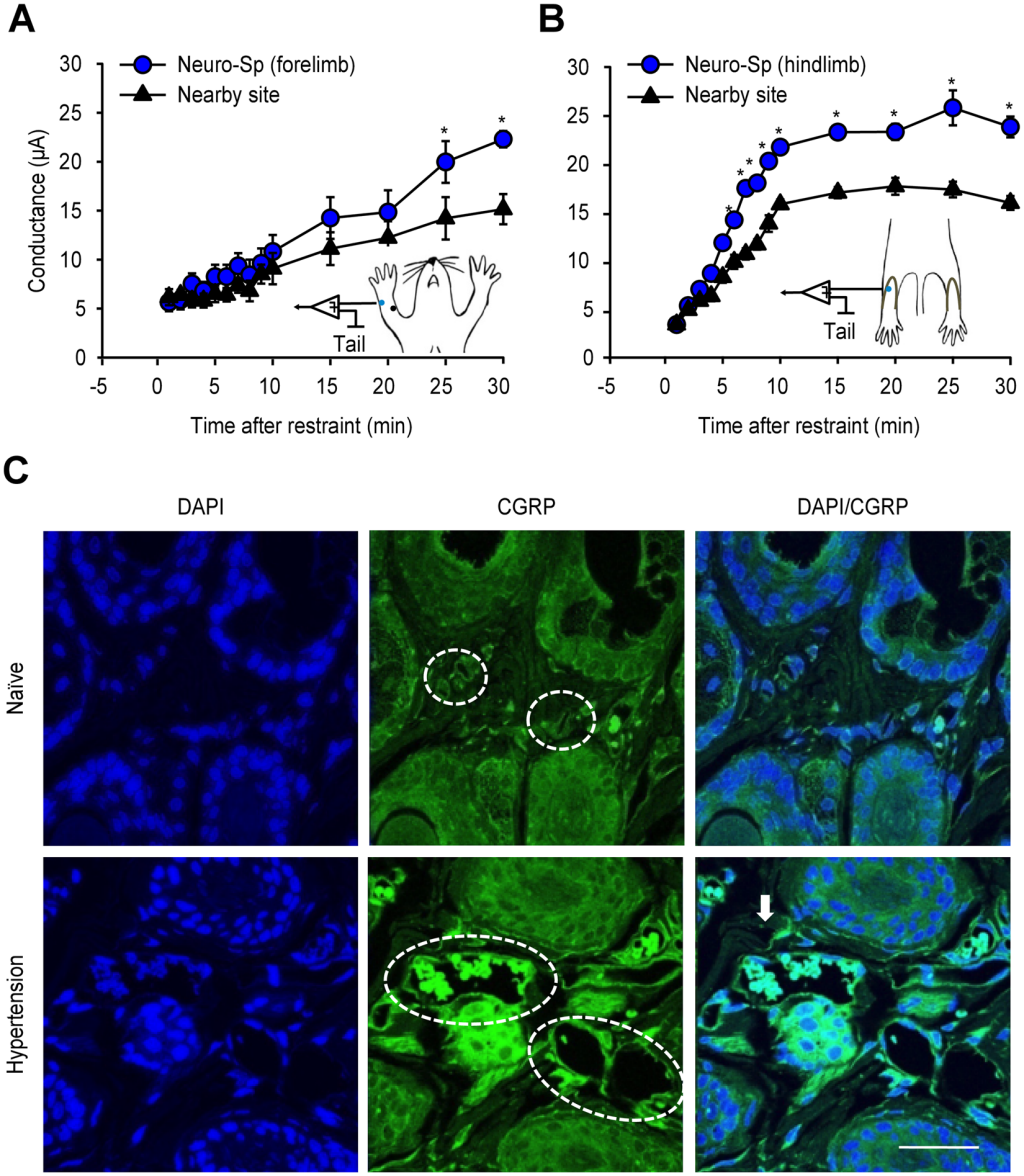
Electrical conductance and CGRP expression in neurogenic spots. (**A**,**B**) Electrical skin conductance. Neurogenic spots revealed higher electrical conductance than nearby sites when measured at neurogenic spots on the forelimb (**A**) or hind paw (**B**) in hypertensive rats. *P < 0.05 vs. Nearby site. (**C**) Increased CGRP expression and vasodilation at neurogenic spots. Note the increased fluorescent intensity of CGRP (green) and markedly dilated microvessels (dotted circle) in the dermis of hypertensive rats compared to naïve rats. Scale bar = 50 µm.

Electrical currents were measured at the neurogenic spots over the wrist or foot in the hypertension model. Skin conductance at a neurogenic spot on the wrist (Fig. 4A) or foot (Fig. 4B), as well as in the adjacent skin, was gradually increased with the development of hypertension following restraint. The levels over the wrist (treatment F(1, 2) = 12.154, P = 0.068, Partial Eta-Squared = 0.854; time F(17, 34) = 36.174, P < 0.001; interaction F(17, 30) = 3.871, P < 0.001, Partial Eta-Squared = 0.686; Fig. 4A) or foot (treatment F(1, 5) = 80.194, P < 0.001, Partial Eta-Squared = 0.941; time F(14, 70) = 190.069, P < 0.001; interaction F(14, 70) = 9.176, P < 0.001, Partial Eta-Squared = 0.647; Fig. 4B) were higher in the neurogenic spots than in the nearby skin. The data showed that the neurogenic spots displayed higher electrical conductance than the surrounding skin.

To determine whether activation of CGRP and vasodilation occurred at neurogenic spots, CGRP immunohistochemistry was performed for the skin over the wrist in the hypertensive rats. Increased fluorescence intensity of CGRP (green) and markedly microvessel dilation (dotted circle) were observed in the dermis over the wrist of the hypertensive rats compared to those of the naïve rats (Fig. 4C).

**Figure 5:**
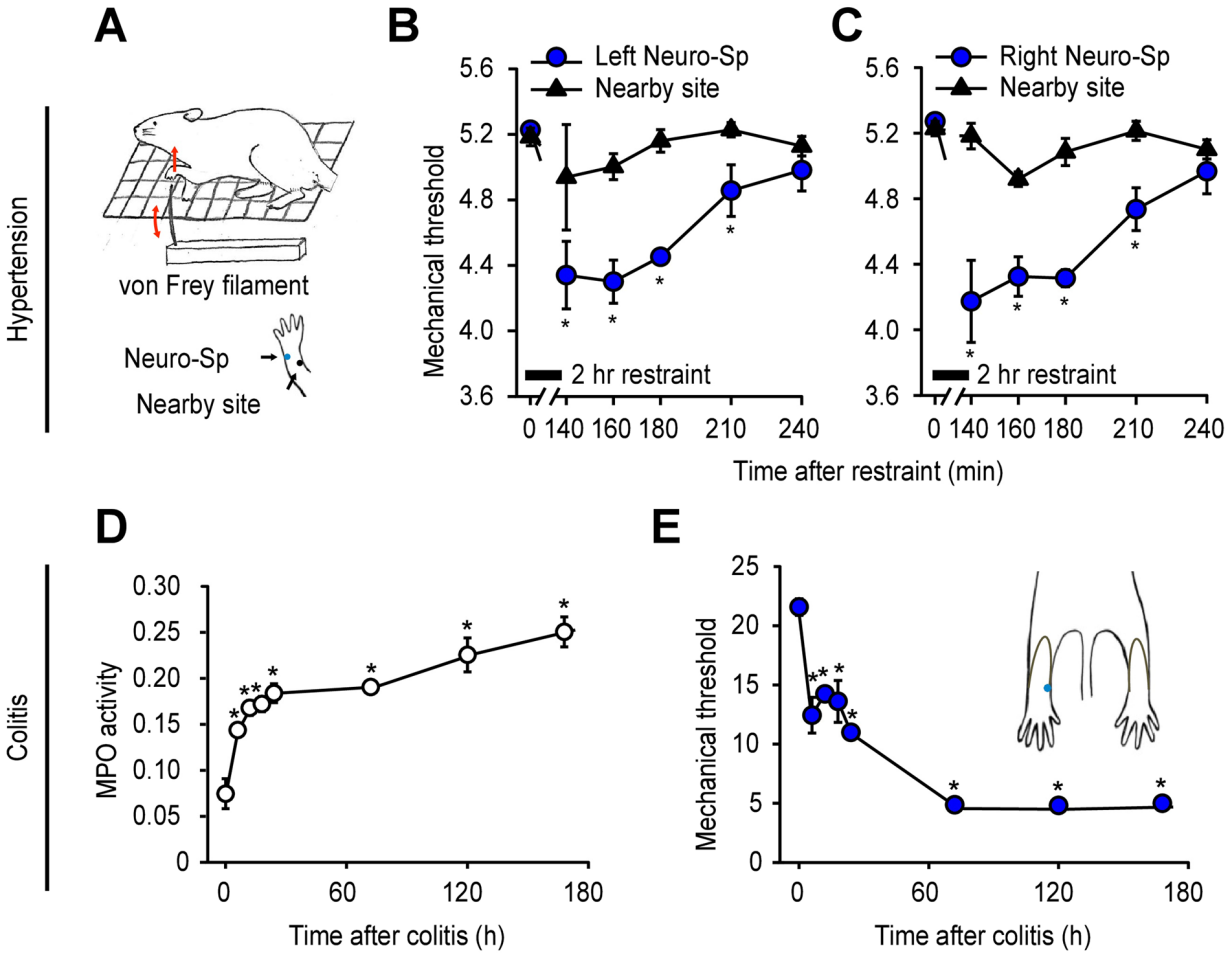
Mechanical sensitivity of neurogenic spots in hypertensive and colitic rats. (**A**–**C**) Mechanical sensitivity of neurogenic spot in hypertensive rats. *P < 0.05 vs. Nearby site. Neurogenic spots on the left (**B**) or right forelimb (**C**) showed lower mechanical thresholds than the nearby sites up to 90 min after the termination of restraint. (**D**,**E**). Correlation between colonic inflammation and mechanical sensitivity of neurogenic spots. Myeloperoxidase (MPO) activity (**D**) and mechanical sensitivity to von Frey hair filament stimulation (**E**) were measured in the colitis rats over 7 days after the induction of colitis.

To see whether neurogenic spots become hypersensitive in hypertension or colitis models, cutaneous mechanical sensitivity was measured at neurogenic spots by measuring paw withdrawal thresholds with a von Frey filament (Fig. 5A). In the hypertension model, the mechanical thresholds of the neurogenic spots over the left wrist (treatment F(1, 4) = 45.418, P = 0.003, Partial Eta-Squared = 0.918; time F(5, 20) = 5.322, P = 0.003; interaction F(5, 20) = 3.879, P = 0.013, Partial Eta-Squared = 0.492; Fig. 5B) or right wrist (treatment F(1, 4) = 35.268, P = 0.004, Partial Eta-Squared = 0.898; time F(5, 20) = 13.336, P < 0.001; interaction F(5, 20) = 9.034, P < 0.001, Partial Eta-Squared = 0.853; Fig. 5C) were significantly decreased, peaking at 20 min after the 2-h restraint and slowly recovering to baseline over 240 min. In the colitis rats, mechanical thresholds at the neurogenic spots over the hind paw (Fig. 5E) were inversely correlated with an increase of MPO activity—an indicator of colitis (Fig. 5D) (Pearson correlation coefficient, −0.914; P = 0.0015). This result showed an increase in sensitivity of neurogenic spots under pathological conditions.

**Figure 6:**
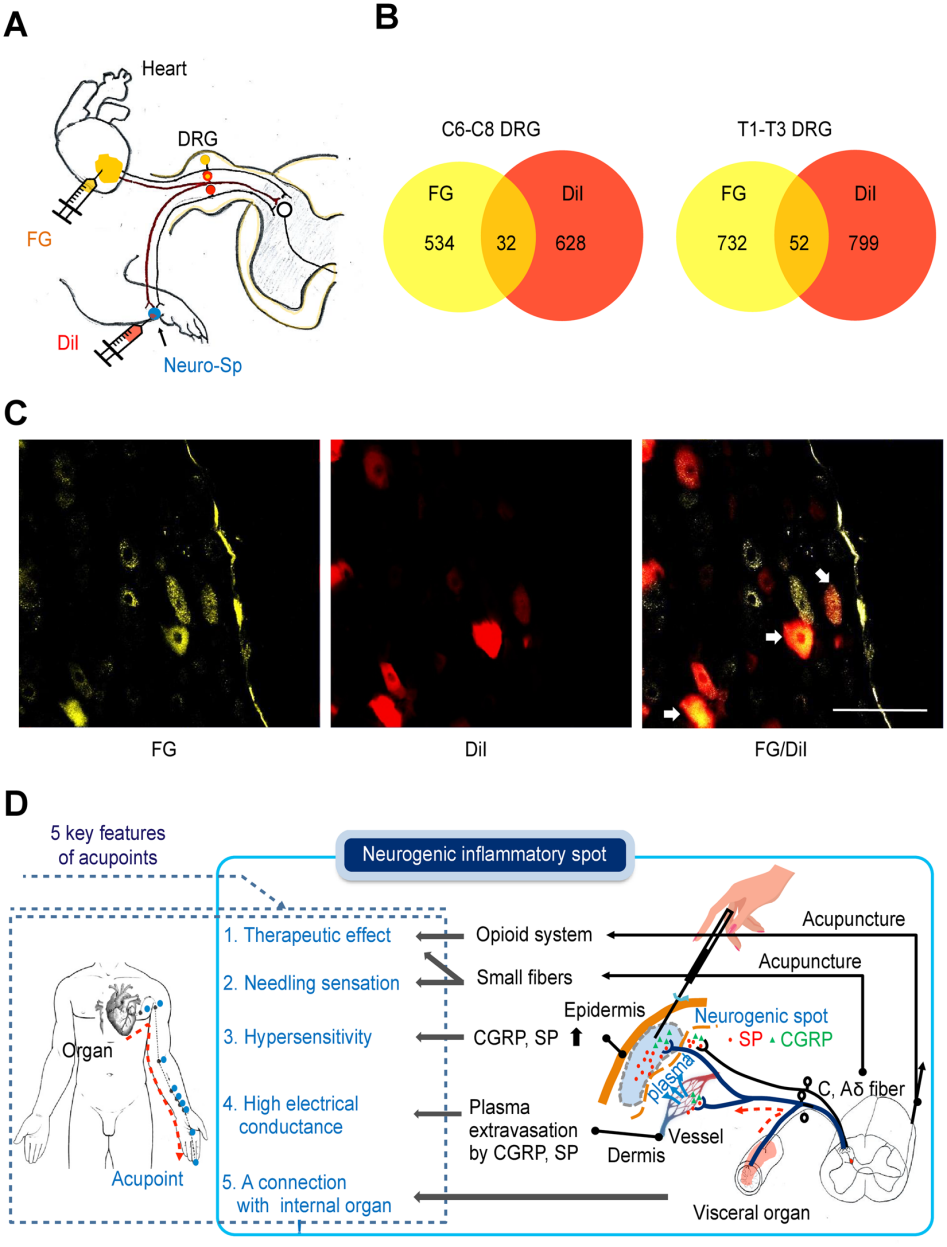
Convergence of afferents from neurogenic spots and the heart on DRGs. (**A**–**C**) When two different retrograde tracers, FG (yellow) and DiI (red), were injected at heart and neurogenic spots, respectively, DiI + FG-double labelled cells (orange) were found in C6-T3 DRG neurons. (**D**) A diagrammatic representation of the proposed hypothesis that neurogenic inflammatory spots are identical to traditional acupoints associated with internal organs. Visceral nociceptive signals produce neurogenic inflammatory spots, caused by the local release of the neuropeptides CGRP and SP from small diameter afferents. The neuropeptides in the skin cause mechanical hypersensitivity and plasma extravasation, yielding high electrical conductance. The activation of small fibres in neurogenic spots can produce needling (Deqi) sensations and therapeutic effects on the associated visceral organs via the endogenous opioid system. Neurogenic spots on the skin are linked to the internal organs. The features of neurogenic spots overlap with those of acupoints. Left panel: an example chart of heart-associated meridian and acupoints in humans.

To show connectivity of the neurogenic spot with the internal organ, two different retrograde tracers, DiI and FG, were injected into neurogenic spots on the wrist and apex of the heart, respectively, in the hypertensive rats (Fig. 6A). Approximately 6% (5.7% for C6-C8 and 6.6% for T1-T3) of FG-labelled DRG neurons exhibited double labelling with DiI (Fig. 6B,C), indicating the convergence of visceral and somatic afferents on the same sensory neurons.

## Discussion

Consistent with the physiological characteristics of acupoints^7–9,13^, our study showed that neurogenic spots caused by activation of somatic afferents during visceral disorders: (1) are found most frequently in the same anatomical location as traditional acupoints, (2) reveal high electrical conductance, and (3) show mechanical hypersensitivity. The linkage of neurogenic spots on the skin to internal organs was verified in a hypertensive rat model by showing that several sensory neurons exhibit branching to both the skin and the heart. Lastly, stimulation of neurogenic spots produces acupuncture effects mediated by the endogenous opioid system. The present study suggests that acupoints associated with internal organs may be identical to neurogenic inflammatory spots occurring on the skin that are associated with visceral disorders.

### Neurogenic spots anatomically correspond to traditional acupuncture points

Noxious signals from viscera frequently produce referred pain at somatotopically distinct body surfaces, which is generally attributed to viscerosomatic convergence at the spinal cord segments^15^. In the somatic area of referred pain, neurogenic inflammatory spots are found and can be easily visualized by intravenously injecting Evans blue dye^17^. In the present study, most of the neurogenic spots in the hypertensive rats were found in the dermatome, which is innervated by the same spinal segments (C8-T2) that innervate the heart^22^, and 67% of those spots matched with acupoints, such as PC6, PC7, and HT7. The acupoints are prescribed most frequently for cardiac disorders^1^ or have been shown to be effective in cardiovascular disorders. In support of these findings, multiple studies have shown that acupuncture stimulation at PC6 can improve or treat cardiovascular disorders, including hypertension, myocardial infarction or hypotension, in humans or experimental animals^23,24^. In contrast, colitis rats revealed neurogenic spots mainly in the hind paw and frequently in the lower back, thighs or tail, which is consistent with our previous study^25^. The spots were found in the dermatome corresponding to the spinal cord sections L2-S2, as mapped by electrical stimulation of C-fibres in the spinal nerve in rats^26^. Given that the nerves innervating the colon project to the T13-L2 and L6-S1 spinal cord segments and those of hind paw primarily enter at the L4-L5 spinal segments, there seems to be no overlapping spinal segments between the colon and hind paw. However, the spinal neurons in nearby segments, including L4-L5, are known to be activated during colon inflammation^27^, providing the neuroanatomical basis for the occurrence of the neurogenic spots in the hind paw in the colitis rats. The spots activated by colitis also corresponded to acupoints such as SP4, ST44, and BL66, which are points commonly used in acupuncture clinics for the treatment of gastrointestinal disorders^1^. These results indicate that many neurogenic spots are found in the dermatome of segmentally related organs and correspond to the location of acupoints commonly used in acupuncture medicine for the treatment of visceral disorders. It is noted that the number and distribution of neurogenic spots in the skin varied among rats with hypertension or colitis (Fig. 1 and Supplementary Table S1). In line with our findings showing DRGs double-labelled with DiI and FG (Fig. 5B), DRG neurons with dichotomized afferents innervating both skin and viscera have been reported^28^ and are considered as a mechanism of referred visceral pain^17^. However, the degree of dichotomized afferents varies considerably among subjects^29^. This variability may be linked to the variations in neurogenic spots among rats in this study.

### Stimulation of neurogenic spots produces acupuncture-like effects via the endogenous opioid system

An important characteristic of acupoints is that their stimulation should produce therapeutic effects on the associated visceral disorders^1,2^. The present study showed that acupuncture at neurogenic spots attenuated the development of hypertension or colitis. However, these effects were not reproduced by acupuncture stimulation of either a nearby site 3–5 mm away from the neurogenic spots or a non-neurogenic acupoint, PC4, which is used in acupuncture clinics for the treatment of hypertension^1^. These results suggest that when stimulated with acupuncture, neurogenic spots generate therapeutic effects on the associated visceral disorders and that neurogenic spots may represent truly effective acupoints rather than conventional acupoints, which is supported by our previous study showing that GV1, an acupoint between the tail base and the anus, reveals neurogenic inflammation in colitis and has therapeutic effects on colitis when acupuncture is applied^25^. Our data are consistent with previous animal studies showing that needling at sham or inactive acupoints, located 3–5 mm from active (verum) acupoints, is ineffective or less effective than needling at active acupoints^30,31^. It is known that sensory nerve endings are distributed unevenly over the body and acupoints have a higher density of sensory nerve endings than surrounding areas^32^. Moreover, previous studies have suggested that active acupoints are associated with tissues where the sensory nerve endings are sensitized by neurogenic inflammatory mediators^33,34^. Given that the sensitized sensory nerve endings are more sensitive to external stimuli than intact sensory nerves, active acupoints with a high density of sensory nerve endings are sensitized by neurogenic inflammatory mediators under pathological conditions. Therefore, stimulation of these sensitive points would generate therapeutic effects by reaching physiological thresholds, compared with stimulation of normal surrounding tissues, such as sham or inactive acupoints. However, in systemic reviews of clinical trials, stimulation of sham acupoints can elicit similar effects as stimulation of verum acupoints in human subjects^35^. Conversely, experimental animal studies have consistently reported the specific effects of active acupuncture are not produced by stimulation of sham acupoints in awake or anesthetized rats^30,31^. The positive effects of sham acupoints in humans may be associated with placebo responses^36^ or inadequate selection of sham acupoints that are several millimeters apart from active acupoints but still located in the effective zone of active acupoints. If the present strategy can be applied to identify active acupoints in human subjects, it may help assess the validity of acupoint specificity by differentiating active acupoints from inactive (sham) acupoints.

It is known that stimulation of small diameter (C and Aδ) nerve fibres during acupuncture treatment generates a sensation (called “*Deqi*”) and acupuncture effects^9,13^. The major part of the “Deqi” sensations, which manifest as pressing, numbing, dull, cold and throbbing sensations, is elicited by small fibre stimulation in acupoints. As shown by enhanced CGRP expression (Fig. 4C), the neurogenic spots display small afferent fibre activation^17^, which may evoke or mediate the acupuncture-specific “Deqi” sensations during needling. Furthermore, the present study showed that small fibre activation by injecting capsaicin or mustard oil into neurogenic spots attenuated the development of hypertension, suggesting that the effects of neurogenic spots are generated through the activation of small diameter nerve fibres. Similarly, a previous study revealed that traditional or electrical acupuncture applied to the PC5-6 acupoints activate small fibres to evoke cardiovascular effects^37^. Taken together, these findings suggest that neurogenic spots may require the activation of small afferent fibres to produce therapeutic effects in a similar manner as traditional acupuncture.

Cumulative evidence suggests that various effects of acupoint stimulation are mediated through the endogenous opioid system^11,38,39^. Stimulation of effective acupoints exerts strong influences on endogenous opioids in the brain and the released opioids play critical roles in producing various acupuncture effects. The endogenous opioid system is considered a main pathway for the onset of various acupuncture effects^40^. A previous study showed that acupoint stimulation activates opioid receptors in the RVLM, a central site for the regulation of blood pressure^41^, and suppresses visceral reflex stimulation-induced hypertension^42^. Similarly, in the present study, the anti-hypertensive effects of neurogenic spot stimulation were abolished by pretreatment of the RVLM with naloxone and replicated by the administration of morphine. Our previous study revealed that acupuncture at the neurogenic GV1 point alleviates diarrhea and colonic inflammation, indicated by enhanced MPO levels, via the endogenous opioid system, and suggested that the increased opioid peptides would generate anti-inflammatory effects by decreasing the enhanced levels of MPO and pro-inflammatory cytokines in the colon^43,44^. These findings suggest that the effect of the stimulation of a neurogenic spot recruits the endogenous opioid system in a similar manner as acupoint stimulation.

### Neurogenic spots reveal the same physiological features as acupuncture points

Acupoints are frequently described as having electrically distinct properties, including high electrical conductance and potential, low impedance and resistance, and increased capacitance^8,9^. The physiological mechanisms underlying these electrical properties in acupoints have not been elucidated. In the present study, the electrical conductance at neurogenic spots was increased with the development of hypertension and higher in neurogenic spots than in the nearby skin, suggesting that neurogenic spots share electrical properties with acupoints. The present study also showed increased CGRP expression, markedly microvessel dilation in the dermis of neurogenic spots and plasma extravasation (as detected by Evans blue dye). CGRP released by activation of small fibre sensory nerve terminals causes neurogenic inflammation in the skin by activating vasodilation, axon reflex flare, and microvascular plasma extravasation^18,45^. CGRP has been reported to be involved in the peripheral mechanism of acupuncture effects^46^. Thus, we suggest that the vasodilation and plasma extravasation by local tissue release of CGRP increases skin moisturization and the sub-skin tissue water content, increasing electrical conductance at neurogenic spots, which may underlie the electrical properties in acupoints.

It is generally accepted that acupoints become sensitive under pathophysiological conditions of internal organs^5–7^. Chae, *et al*.^6^ reported the increased sensitivity of the acupoint SP6, which is clinically used for reproductive disorders^1^ in women undergoing premenstrual syndrome. In a clinical study, patients with gastric ulcers or gastritis showed significantly decreased pressure-pain thresholds at the disease-related acupoints compared to healthy subjects^5^. In the present study using the von Frey method, the mechanical thresholds of neurogenic spots were decreased with the development of hypertension and colitis, indicating the increased mechanical sensitivity of neurogenic spots. Because the neuropeptides CGRP and SP are released during neurogenic inflammation and induce mechanical and thermal hyperalgesia in the skin^47,48^, the increased sensitivity of neurogenic spots may be attributed to enhanced release of CGRP, as observed in the CGRP immunohistochemistry results. This result suggests that tenderness (an increase in sensitivity) of neurogenic spots under pathological conditions overlaps with the characteristic of traditional acupoints.

TCM describes that each acupoint is linked to a specific internal organ, particularly the acupoints lying along the Heart (HT) or Pericardium (PC) meridians (i.e., PC6, PC7 and HT7) on the medial aspect of the arm, which are internally connected with the heart^1^. The linkage of neurogenic spots on the skin to internal organs was identified by our retrograde study showing convergent DRG neurons innervating both the heart and the neurogenic spots. This finding is in agreement with that of a previous study showing that 7–14% of DRGs from the cardiac area labelled with true blue are simultaneously are labelled with nuclear yellow injected into the ulna nerve in rats^28^. The connection between acupoints and the internal organs in TCM may be explained by the viscero-somatic convergence that occurs at neurogenic spots.

In conclusion, the present study suggests that traditional acupoints associated with internal organs represent one form of neurogenic inflammation occurring on the skin that is associated with visceral disorders.

## Methods

### Animals

Male Sprague-Dawley rats (weight 270–320 g, Daehan Animal, Korea) were used. All animal experiments were carried out in accordance with the National Institutes of Health Guide for the Care and Use of Laboratory Animals and approved by the Institutional Animal Care and Use Committees at Daegu Haany University and Kyung Hee University.

### Chemicals

Evans blue dye (1 ml/kg; 50 mg/ml in saline, Sigma-Aldrich, USA), capsaicin (a transient receptor potential vanilloid 1 (TRPV1) agonist, 0.05%, 10 μl/loci; Sigma-Aldrich) in a vehicle consisting of 10% alcohol and 10% Tween 80 in saline, mustard oil (a transient receptor potential A1 (TRPA1) agonist; 20% in mineral oil, 10 μl/site; Sigma-Aldrich), morphine (10 mg/kg; Jeil Pharmaceutical, Korea; an opioid agonist), naloxone (a non-specific opioid antagonist, 10 nM in saline; Sigma-Aldrich), Fluoro-Gold (FG; 5% in saline, 10 μl/site, Fluorochrome, USA), and DiI (FAST DiI; DiIΔ^9,12^-C_18_(3), CBS (1,1′-dilinoleyl-3,3,3′,3′-tetramethylindocarbocyanine, 4-chlorobenzenesulfonate); 10 μl/site; 5 mg in 0.1 ml methanol; Thermo Scientific, USA) were used.

### Immobilization-induced hypertension and measurement of blood pressure

Hypertension was induced by using a cone-shaped plastic bag, as described previously^21^. Systolic blood pressure was measured non-invasively with a tail cuff blood pressure monitor (Model 47, IITC). Briefly, the restrained rats were placed in a chamber kept at 27 °C, and an occluding cuff and pneumatic pulse transducer were positioned on the base of the rat’s tail. A programmed electrosphygmomanometer (Narco Bio-Systems Inc., USA) was inflated and deflated automatically and the tail cuff signals from the transducer were automatically collected every 10 min using an IITC apparatus (Model 47, IITC Inc., USA). The mean of two readings was taken at each blood pressure measurement. The rats that had sustained systolic blood pressure over 150 mmHg were considered to have developed hypertension, as defined elsewhere^49^.

### Colitis

Experimental colitis was induced using trinitrobenzene sulfonic acid (TNBS; Sigma-Aldrich) in ethanol, as described previously^50^. Following a 12-h fast, rats were anaesthetized with a combination of oxygen (1 l/min) and 3.5% isoflurane. An intragastric tube was inserted rectally into the colon so that the tip was placed 8 cm proximal to the anus. Thereafter, 0.60 ml trinitrobenzene sulfonic acid (TNBS 40 mg, Sigma-Aldrich) in 0.25 ml of 50% ethanol (v/v) was instilled into the lumen of the colon. The animals were then maintained in a head-down position for approximately 60 sec to prevent leakage. In the control group, a similar volume of physiological saline was injected into the colon.

### Visualization of neurogenic spots in the skin by injection of Evans blue dye

Neurogenic spots were visualized by injecting Evans blue dye into male Sprague-Dawley rats as described previously^17^. Briefly, the distal portion of the tail was dipped in 40 °C warm water for at least for 30 sec. Evans blue dye (50 mg/kg, 50 mg/ml saline) was injected via the tail vein with a catheter (26-gauge), and skin colour changes were observed after the injection. The blue-dyed spots on the skin were sketched using body charts, photographed and compared with a human acupoint chart, based on the transpositional method, which locates acupoints on the surface of animal skin corresponding to the anatomic site of human acupoints^19^. Anatomical locations of neurogenic spots were independently assessed by two acupuncture experts (coauthors) among authors, HY Kim and BH Lee, who have more than 20 years’ experience in animal or human acupuncture, respectively. Evans blue dye was injected immediately after immobilization for the hypertension model and one day after TNBS injection for the colitis model, respectively.

### Acupuncture at neurogenic spots

While the animal was restrained in a cone-shaped plastic bag (hypertension model) or a plastic holder (colitis model), acupuncture needles (0.10 mm in diameter) were inserted 3-mm-deep into the neurogenic spots or non-neurogenic nearby sites 3–5 mm apart from the neurogenic spots. For the hypertension model, a reference needle was inserted 2 mm away from the centre of the neurogenic spot near the wrist, which most frequently showed Evans blue dye leakage in the hypertensive rats, and electrical stimulation was applied to the needles for 10 min (electroacupuncture; EA). Since a previous study showed that electrical stimulation at low frequency effectively reduces hypertension^37^, a similar condition of electrical stimulation (3 Hz, 0.5 mA, 0.1 ms, triangular pulses) was chosen from our electrostimulator with various pre-programmed settings (Han-il Co, Korea) and the needles were stimulated at low frequency for 10 min. In the colitis model, manual acupuncture was applied as performed in our previous studies^44,51^ with slight modification, since our previous studies revealed that repetitive manual acupuncture successfully ameliorates TNBS-induced colitis in rats^44,51^. Each rat was placed in a plastic holder with the hind limb protruding. Needles were then inserted into bilateral neurogenic spots near the base of the 1st metatarsal bone in which Evans blue dye leakage was most commonly found in the colitis rats, and were maintained for 15 min (manual acupuncture; MA).

### Microinjection of naloxone into the rostral ventrolateral medulla (RVLM)

The animals were fixed on a stereotaxic frame in the prone position. For the microinjections into the RVLM, the nose was deflected ventrally so that the dorsal surface of the medulla could be levelled horizontally. A midline incision was then made on the back of the neck to expose and remove the atlanto-occipital membrane. A 1.0-mm burr hole was made 12.72 mm posterior to bregma and 2 mm lateral (right and left) to the midline. A 26-gauge needle on a Hamilton syringe was inserted 10 mm deep into the right and left area. Naloxone was perfused at a constant rate of 0.1 μl/min (CMA 100, microinjection pump; KD Scientific, USA). To verify the injection sites, under pentobarbital anaesthesia, the brain stem was removed, fixed in 4% paraformaldehyde (PFA), cryosectioned and examined under a microscope.

### Macroscopic assessment of colitis

Body weight changes were monitored, and diarrhea scores were rated: (0, normal; 1, mild; 2, watery; 3, bloody diarrhea). Seven days after colitis induction, the distal 8-cm portion of the colon was removed and evaluated with parameters of colon weight/colon length, adhesions (score 0~2), diarrhea (score 0~1), and clinical features (score 0~10) according to the criteria of Bobin-Dubigeon, *et al*.^52^. The collected colon was immediately frozen at −70 °C to analyse myeloperoxydase (MPO) activity and the mRNA expression levels of TNF-α and IL-1β.

### Myeloperoxidase (MPO) activity and mRNA expression of TNF-α and IL-1β in the colon

MPO activity was measured spectrophotometrically with O-dianisidine and hydrogen peroxidase, as described previously^53^. Under pentobarbital anaesthesia, the colon was removed and weighed. The tissue was thawed and homogenized in 4 volumes of 50 mM potassium phosphate buffer (pH = 7). The pellets were again homogenized in 1 volume of 50 mM potassium phosphate buffer containing 1% hexadecyltrimethylammonium bromide (HETAB). The homogenate was subjected to 3 freeze/thaw/sonication cycles. The mixture was incubated at 4 °C for 20 min. The homogenate was centrifuged at 11,500 rpm for 15 min at 4 °C. An aliquot of the homogenate (0.1 ml) was added to 2.4 ml of 0.5% O-dianisidine and 0.5 ml of 0.0029% hydrogen peroxidase. The mixture was incubated at 25 °C for 10 min, and then 0.5 ml 0.1% NaN_3_ was added. The reaction was measured using a spectrometer (Ultrospec 2100pro, GE Healthcare Bio-sciences, USA) at 460 nm.

The mRNA expression of TNF-α and IL-1β in the colon was determined according to previously described methods from our laboratory^54^. Briefly, RNA extraction and cDNA synthesis were conducted. The primer sequences used for semi-quantitative PCR were as follows: **GAPDH**, (forward) 5′-ATC CCA TCA CCA TCT TCC AG-3′ and (reverse) 5′-CCT GCT TCA CCA CCT TCT TG-3′; **IL1-β**, (forward) 5′-GGC ATA ACA GGC TCA TCT GG-3′ and (reverse) 5′-CAT CAT CCC ACG AGT CAC AG-3′; **TNF-α**, (forward) 5′-GTC GTA GCA AAC CAC CAA GC-3′ and (reverse) 5′-GAC TCC AAA GTA GAC CTG CCC-3′. The PCR products were visualized on a 1% agarose gel with GelRed^®^ (Biotium, USA). The band intensities were quantified using an image analysis system (i-Max, CoreBio System, Korea) and normalized to that of GAPDH.

### Measurement of skin electrical conductance

Skin electrical currents were measured using a GSR AMP device (Model FE116, ADInstrument, Australia). Evans blue dye was injected 10 min after the initiation of restraint. The negative electrode was used for skin electrical current on neurogenic spots or adjacent areas, while the positive electrode was attached to the surface of the tail. Electrical currents were measured up to 240 min after the initiation of restraint.

### Immunohistochemistry for CGRP

One hour after restraint, skin samples were taken from the wrist, which most frequently showed Evans blue dye leakage in the hypertensive rats. The skin samples were paraffin-embedded, sectioned (5 μm), and incubated with primary antibodies (1:500, mouse anti-CGRP; Chemicon, USA; Cat# MAB317, RRID:AB_2275141), followed by incubation with secondary antibody (1:500, Alexa Fluor 488-conjugated donkey anti-mouse IgG antibody, Thermo Scientific; Cat# A11055, RRID:AB_142672). The sections were mounted on gelatine-coated slides, air-dried, and coverslipped with a mounting medium containing DAPI (Vector, Cat# H-1500). The sections were examined using a laser-scanning confocal microscope (LSM700, Carl Zeiss, Germany).

### Mechanical sensitivity

Mechanical sensitivity at neurogenic spots was determined by the withdrawal response of the forelimb (hypertension model) or hind limb (colitis model) to the probing of von Frey filaments. Briefly, each animal was placed in a plastic chamber (8.0 × 9.0 × 24 cm) on top of a mesh screen platform. For the hypertension model, mechanical thresholds were assessed before and up to 120 min after termination of the 2-hr restraint by the up-down method^55^ using a set of von Frey monofilaments. The data were plotted using a linear scale in von Frey values. For the colitis model, mechanical thresholds were measured before and up to 7 days after colitis induction using an electronic von Frey anaesthesiometer (IITC) and plotted as gram force.

### Colocalization of Fluoro-Gold (FG) and DiI in dorsal root ganglion neurons (DRGs)

Two different neurotracers, FG and DiI, were injected into the apex of the heart and a neurogenic spot near the wrist, respectively. Briefly, under isoflurane anaesthesia, Fast DiI (5%, 10 μl) was injected into a neurogenic spot near the wrist using a Hamilton syringe with a 27-gauge needle. To inject FG (5%, 10 μl) into the heart, the chest wall was opened at the 4^th^ or 5^th^ left intercostal space, the pericardial sac was incised using fine forceps, and FG was slowly injected into the left ventricular muscle of the heart. The chest wall and skin were closed aseptically and the antibiotic gentamycin (2 mg/kg, i.m.) was administered. Two weeks later, the rats were anaesthetized with pentobarbital sodium (90 mg/kg, i.p) and perfused to isolate the dorsal root ganglion (DRG) neurons at C6-C8 and T1-T3. The DRGs were post-fixed in 4% buffered PFA for 2 h, immersed in 30% sucrose overnight and cryosectioned at 30 μm. The cryosections were then mounted on gelatine-coated glass slides. The DRGs labelled by DiI and/or FG were imaged under a confocal laser scanning microscope (LSM700, Carl Zeiss, Germany).

### Data Analysis

All data are presented as the mean ± standard error of the mean (SEM) and analysed by one- or two-way repeated-measures analysis of variances (ANOVAs), followed by post hoc testing using the Tukey method or t-test where appropriate. Pearson correlation coefficients were calculated to assess the relationships between MPO activity and mechanical threshold. Statistical significance was considered at P < 0.05.

## Electronic supplementary material

Supplementary Table S1
