# Supplementary material for: Acupuncture points can be identified as cutaneous neurogenic inflammatory spots
Source: Sci Rep. 2017 Nov 9;7:15214. doi: 10.1038/s41598-017-14359-z (PMC5680247; doi:10.1038/s41598-017-14359-z)
Supplement: Supplementary file 1 — Supplementary Table S1 [file 41598_2017_14359_MOESM1_ESM.pdf]

<Supplementary Materials>

Acupuncture points can be identified as cutaneous neurogenic inflammatory spots

Authors: Do-Hee Kim, Yeonhee Ryu, Dae Hyun Hahm, Boo Yong Sohn, Insop Shim, O Sang Kwon, Suchan Chang, Young Seob Gwak, Min Sun Kim, Jae Hyo Kim, Bong Hyo Lee, Eun Young Jang, Rongjie Zhao, Jin Mo Chung, Chae Ha Yang, Hee Young Kim

Supplementary Table S1. Number of neurogenic spots corresponding to known acupoints in the hypertension and colitis models. FL=forelimb, HL=hind limb

Model	Hypertension		Colitis	
	Location of Neuro-Sp	Number of Neuro-Sp (n = 18 rats)	Location of Neuro-Sp	Number of Neuro-Sp (n = 13 rats)
Acupoints	PC6 (FL)	28	SP4 (HL)	12
	PC7 (FL)	24	ST44 (HL)	7
	HT7 (FL)	22	BL66 (HL)	4
	SI3 (FL)	5	BL25 (HL)	3
	PC4 (FL)	4	BL29 (HL)	1
	LU9 (FL)	2	BL64 (HL)	2
	LR3 (HL)	2	BL65 (HL)	1
	BL54 (HL)	1	GB32 (HL)	1
			GV1	2
			KD14 (HL)	1
			ST25 (HL)	3
			ST27 (HL)	1
			ST31 (HL)	1
			ST35 (HL)	1
			ST36 (HL)	3
			ST42 (HL)	1
			ST43 (HL)	1
			ST44 (HL)	1
	Subtotal	88	Subtotal	46
Non-Acupoints	fore-digits (FL)	5	abdomen	3
	left heel (HL)	16	scrotum	2
	right heel (HL)	15	tail	2
	hindpaw pad (HL)	7	tail base	3
			left heel (HL)	2
			right heel (HL)	3
	Subtotal	43	Subtotal	15
Total		131		61
